# Pre-therapy PET-based voxel-wise dosimetry prediction by characterizing intra-organ heterogeneity in PSMA-directed radiopharmaceutical theranostics

**DOI:** 10.1007/s00259-024-06737-3

**Published:** 2024-05-09

**Authors:** Song Xue, Andrei Gafita, Yu Zhao, Lorenzo Mercolli, Fangxiao Cheng, Isabel Rauscher, Calogero D’Alessandria, Robert Seifert, Ali Afshar-Oromieh, Axel Rominger, Matthias Eiber, Kuangyu Shi

**Affiliations:** 1 Dept. Nuclear Medicine, Bern University Hospital, University of Bern, Bern, Switzerland; 2 Dept. Nuclear Medicine, Technical University of Munich, Munich, Germany; 3 Chair for Computer Aided Medical Procedures, School of Computation, Information and Technology, Technical University of Munich, Munich, Germany; 4 Department of Biomedical Imaging and Image-Guided Therapy, Division of Nuclear Medicine, Medical University of Vienna, Vienna, Austria; 5 Bavarian Cancer Research Center, (BZKF), Erlangen, Germany

**Keywords:** Radiopharmaceutical therapy, [^177^Lu]Lu-PSMA I&T, Dosimetry, Deep learning, Intra-organ heterogeneity

## Abstract

**Background and objective:**

Treatment planning through the diagnostic dimension of theranostics provides insights into predicting the absorbed dose of RPT, with the potential to individualize radiation doses for enhancing treatment efficacy. However, existing studies focusing on dose prediction from diagnostic data often rely on organ-level estimations, overlooking intra-organ variations. This study aims to characterize the intra-organ theranostic heterogeneity and utilize artificial intelligence techniques to localize them, i.e. to predict voxel-wise absorbed dose map based on pre-therapy PET.

**Methods:**

23 patients with metastatic castration-resistant prostate cancer treated with [^177^Lu]Lu-PSMA I&T RPT were retrospectively included. 48 treatment cycles with pre-treatment PET imaging and at least 3 post-therapeutic SPECT/CT imaging were selected. The distribution of PET tracer and RPT dose was compared for kidney, liver and spleen, characterizing intra-organ heterogeneity differences. Pharmacokinetic simulations were performed to enhance the understanding of the correlation. Two strategies were explored for pre-therapy voxel-wise dosimetry prediction: (1) organ-dose guided direct projection; (2) deep learning (DL)-based distribution prediction. Physical metrics, dose volume histogram (DVH) analysis, and identity plots were applied to investigate the predicted absorbed dose map.

**Results:**

Inconsistent intra-organ patterns emerged between PET imaging and dose map, with moderate correlations existing in the kidney (*r* = 0.77), liver (*r* = 0.5), and spleen (*r* = 0.58) (*P* < 0.025). Simulation results indicated the intra-organ pharmacokinetic heterogeneity might explain this inconsistency. The DL-based method achieved a lower average voxel-wise normalized root mean squared error of 0.79 ± 0.27%, regarding to ground-truth dose map, outperforming the organ-dose guided projection (1.11 ± 0.57%) (*P* < 0.05). DVH analysis demonstrated good prediction accuracy (R^2^ = 0.92 for kidney). The DL model improved the mean slope of fitting lines in identity plots (199% for liver), when compared to the theoretical optimal results of the organ-dose approach.

**Conclusion:**

Our results demonstrated the intra-organ heterogeneity of pharmacokinetics may complicate pre-therapy dosimetry prediction. DL has the potential to bridge this gap for pre-therapy prediction of voxel-wise heterogeneous dose map.

**Supplementary Information:**

The online version contains supplementary material available at 10.1007/s00259-024-06737-3.

## Introduction

Radiopharmaceuticals therapy (RPT) is a contemporary approach to radiation oncology, which aims to deliver the maximally destructive radiation dose via cancer-targeting radiopharmaceutical [1]. In the last decades, advances in molecular biology and pharmacology have furnished a wide range of radioactive substances targeting receptors in cancer cell [2]. Compared to traditional external beam radiotherapy (EBRT), RPT delivers radiation dose more extensively to the intended target tissues and has consequently already proven itself to be effective for the treatment of several metastatic or unresectable cancers through systematic and rationalized administration of the radiopharmaceutical [3].

However, concerns have been raised about the risk of inadequate balance between therapeutic dose and side effects in RPT. The European Council Directive (2013/59 Euratom) mandates that RPT treatments should be planned according to the optimal radiation dose tailored for individual patients, as has long been the case for EBRT [4]. The essential requirement of RPT treatment planning is to estimate the absorbed dose in advance of therapy [5]. The recent European Association of Nuclear Medicine (EANM) procedure guidelines for [^177^Lu]Lu-PSMA re-emphasized the value of dosimetry and iterated that exposures of target volumes are to be individually planned and verified.

Theranostics is a unique technology wherein the evaluation of therapeutic agent distribution before treatment informs the treatment protocol [6]. In the context of PSMA RPT, this involves the pre-therapy positron emission tomography (PET) imaging (e.g. [^68^Ga]Ga-PSMA) to determine eligibility for [^177^Lu]Lu-PSMA treatment [7]. Furthermore, post-therapy SPECT/CT serves to estimate tracer distribution and enable dosimetry, facilitating the determination of radiation dose for specific entities such as an organ, tumor, or even a single voxel [8].

Numerous studies have embraced the concept of theranostics to predict dosimetry in RPT. One extensively explored approach involves physiologically based pharmacokinetic (PBPK) models, which elucidate the fundamental principles underlying the uptake process of pharmaceuticals, including radio-labeled ligands for PET imaging and RPT [9]. For example, individualized PBPK model parameters can be derived by pre-therapy PET/CT activity concentrations, planar scintigraphy, and tumor volumes, allowing for the individualization of [^177^Lu]Lu-PSMA-I&T therapy [10]. Artificial intelligence (AI) in medicine has burgeoned over the past decade, with machine learning (ML) particularly holding promise for pre-therapy prediction of dosimetry [11]. Nonetheless, both PBPK-based predictions and our previously developed ML approach are limited to organ-level estimations and do not account for intra-organ heterogeneity, which is crucial for assessing organ toxicity during treatment planning. In the context of RPT, localized areas of high absorbed dose within an organ serve as indicators of organ toxicity, rather than the overall organ dose [12]. Therefore, accurately predicting intra-organ heterogeneity before initiating therapy is essential for balancing treatment benefits and risks, as well as optimizing therapeutic outcomes.

To address this issue, voxel-level dosimetry has been proposed for patient-specific dose assessment in tumors and organs-at-risk (OAR), aiming to determine the absorbed dose for each image voxel [13, 14]. Convolutional neural networks (CNNs), a form of deep learning (DL), have emerged as a powerful tool for image synthesis [15] and can be leveraged to predict voxel-wise absorbed dose map from pre-therapy PET imaging.

This study pursues two main objectives: (1) To investigate the voxel-wise correlation between pre-therapy PET and the therapy dosimetry, thereby characterizing the intra-organ heterogeneity; (2) To explore the feasibility of predicting voxel-wise dosimetry before therapy. We propose two strategies for prediction: (1) Organ-dose guided direct projection; (2) Introducing a novel CNN-based framework named 3D RPT DoseGAN to voxel-wise predict dosimetry. Both strategies are designed to bridge the gap between the distributions of pre-therapy PET imaging and post-therapy dose maps. The overarching aim of this work is to enhance treatment planning for RPT by facilitating accurate predictions of voxel-wise dosimetry.

## Materials and methods

### Data acquisition

Retrospectively, 23 metastatic castration-resistant prostate cancer (mCRPC) patients treated with [^177^Lu]Lu-PSMA I&T RPT were included in our study. Only those cycles with [^68^Ga]Ga-PSMA-11 PET/CT directly before the treatment and at least 3 post-therapeutic SPECT/CT dosimetry imaging and planar scintigraphy were selected. Totally, 48 cycles of [^177^Lu]Lu-PSMA I&T were considered for this proof-of-concept study (22 first, 12 s, 6 third, and 8 fourth or further cycles). After application of approximately 7.4 GBq (7.3 ± 0.3 GBq) [^177^Lu]Lu-PSMA I&T, SPECT/CT dosimetry imaging was performed at least between 30 and 150 min, 24 h, and 6–8 days. The institutional review board of the Technische Universitiät München approved this study, and all subjects signed a written informed consent form. More detailed information of patient cohorts can be found in Table 1.

**Table 1:** Demographic information of patients’ data

Pre-therapy PET Tracer	[^68^Ga]Ga-PSMA-11
Pre-therapy PET Dose (MBq)	118.4 ± 25.1
Therapeutic Radiopharmaceutical	[^177^Lu]Lu-PSMA I&T
Therapeutic Dose (GBq)	7.3 ± 0.3
Number of Patients	23
Total Number of Cycles	48
Average Age (Year)	69 ± 7
Average Weight [37]	78.7 ± 9.9
3 Time Points of Post-therapy SPECT/CT	30–150 min, 24 h, 6–8d

### Data preparation

We rigorously calibrated the SPECT images using the well-established approach by Halty [16], incorporating the whole-body planar image and leveraging the Hermes Hybrid Dosimetry 4.0 tool for assistance. Subsequently, the Hermes Hybrid Voxel dosimetry tool was utilized to generate absorbed dose maps based on the sequential SPECT dosimetry imaging. We utilized PMOD (version 4.1) for rigid registration between the CT from the pre-therapy PET/CT and the CT from the first time-point SPECT/CT, which served as the reference for registration during dose map generation, ensuring proper alignment of the PET and dose map. The image size for all the pre-therapy PET/CT, SPECT/CT, and dose map was standardized to 128 × 128 for each slice.

### Investigation of voxel-wise correlation between pre-therapy PET and absorbed dose map

Previous research has demonstrated a correlation between pre-therapeutic standardized uptake value (SUV) and absorbed dose at the organ level [17]. However, it is crucial to investigate this correlation at the voxel level to establish a foundation for accurate voxel-wise prediction. Therefore, we conducted a feasibility study to examine the correlation between pre-therapy PET imaging and dose map. Our investigation encompassed both voxel values and heterogeneity, which were quantified using radiomics features [18], including Gray-Level Run-length Matrix (GLRLM) and Gray-Level Co-occurrence Matrix (GLCM). The correlation was assessed using the Pearson correlation coefficient. All *P*-values were two-sided, and *P* < 0.025 was considered statistically significant.

### Simulation to interpret the relation between pre-therapy PET and therapy dosimetry

**Fig. 1:**
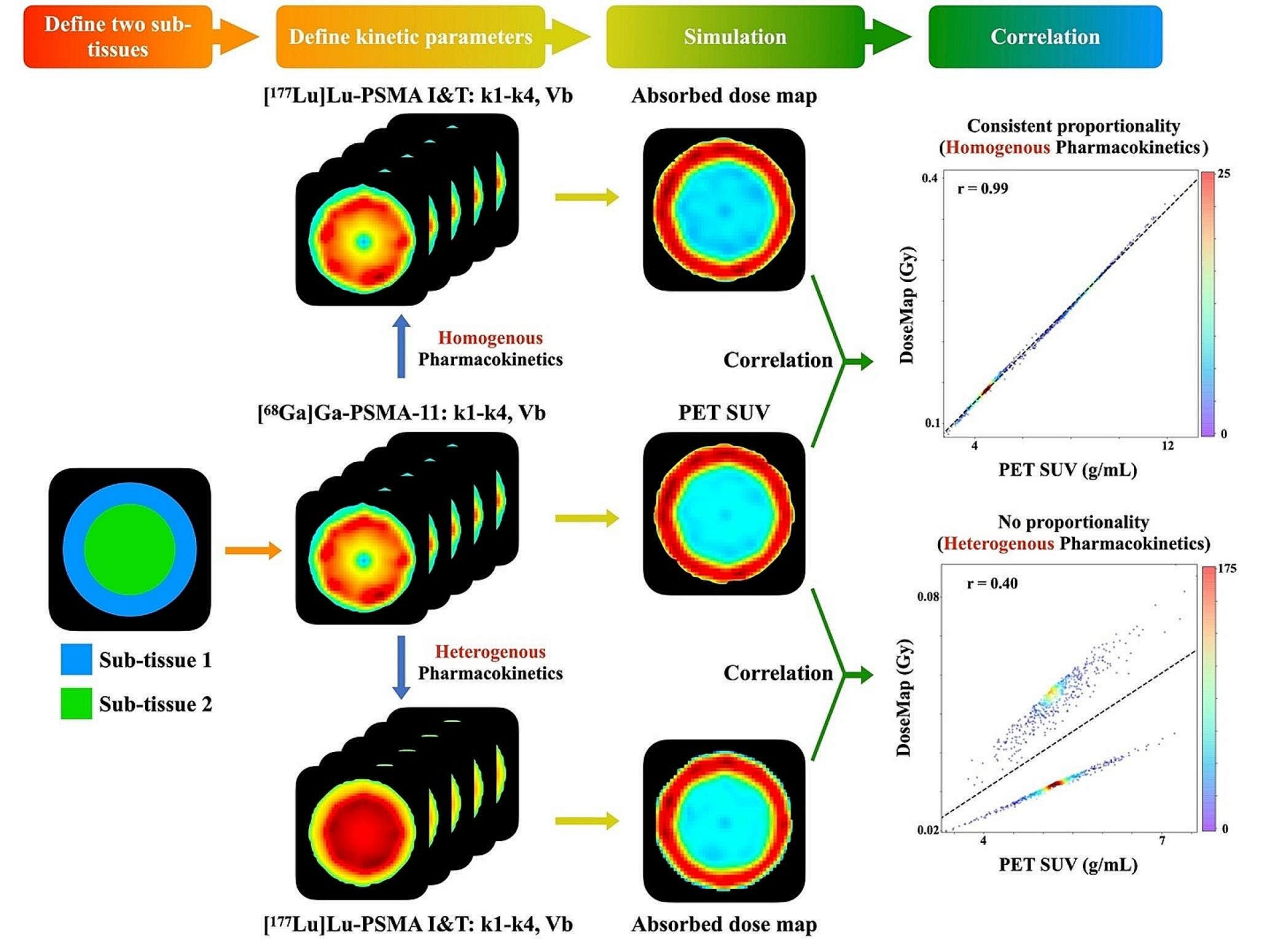
Simulation illustrating the correlation between pre-therapy PET and dose map, underpinned by a two-tissue compartment model. This model imagines a simplified scenario involving two ideal sub-tissues, with a predetermined pharmacokinetic relationship between the PET imaging tracer and the therapeutic compound. This pharmacokinetic relationship can be either homogenous (maintaining constant proportionality) or heterogeneous (without consistent proportionality). Noticeably, a correlation between PET uptake and the accumulated dose becomes apparent solely in scenarios with homogenous pharmacokinetics

A simplified two-tissue compartment modeling [19] were employed to enhance our understanding of the correlation between pre-therapy PET and post-therapy dose map (Fig. 1). The model incorporated two distinct sub-tissues and hypothesized corresponding kinetic parameters was used. Within each sub-tissue, a variation by gradually changing the parameters towards the center was introduced. For each voxel, the parameters were drawn from a normal distribution, using mean and standard deviation values derived from actual patient data.

Kinetic parameters ${\varvec{k}p}_{ij}$ and ${\varvec{k}t}_{ij}$ were considered, where $\varvec{k}p$ and $\varvec{k}t$ signify the pharmacokinetics of the PET tracer and therapy compound respectively. The indices $i=[1-5]$ represent the kinetic parameters $[k1, k2, k3, k4, Vb]$ and $j=[1, 2]$ represent the two sub-tissues. We hypothesized two potential pharmacokinetic relationships between the PET imaging tracer and therapy compound:

1. Homogenous pharmacokinetics: Consistent proportionality exists between the PET imaging tracer and therapy compound across both sub-tissues.

$$\frac{{\varvec{k}p}_{i1}}{{\varvec{k}t}_{i1}}=\frac{{\varvec{k}p}_{i2}}{{\varvec{k}t}_{i2}}$$

2) Heterogenous pharmacokinetics: No proportionality is present between the PET imaging tracer and therapy compound across the sub-tissues.

$$\frac{{\varvec{k}p}_{i1}}{{\varvec{k}t}_{i1}}\ne \frac{{\varvec{k}p}_{i2}}{{\varvec{k}t}_{i2}}$$

For each hypothesis, we applied two-tissue compartment modeling to compute the SUV value at a single time point and the total absorbed dose using the S value of 2.2e-04 mGy/MBq/s for the left kidney cortex [20], derived from ICRP 110 reference phantoms [21]. We assumed the PET imaging tracer as [^68^Ga]Ga-PSMA-11 and the therapy compound as [^177^Lu]Lu-PSMA I&T. Based on these simulations, we examined the correlation between PET SUV and dose map using a heat scatter plot.

### Pre-therapy prediction of absorbed dose map

#### Approach 1: organ-dose guided direct projection

**Fig. 2:**
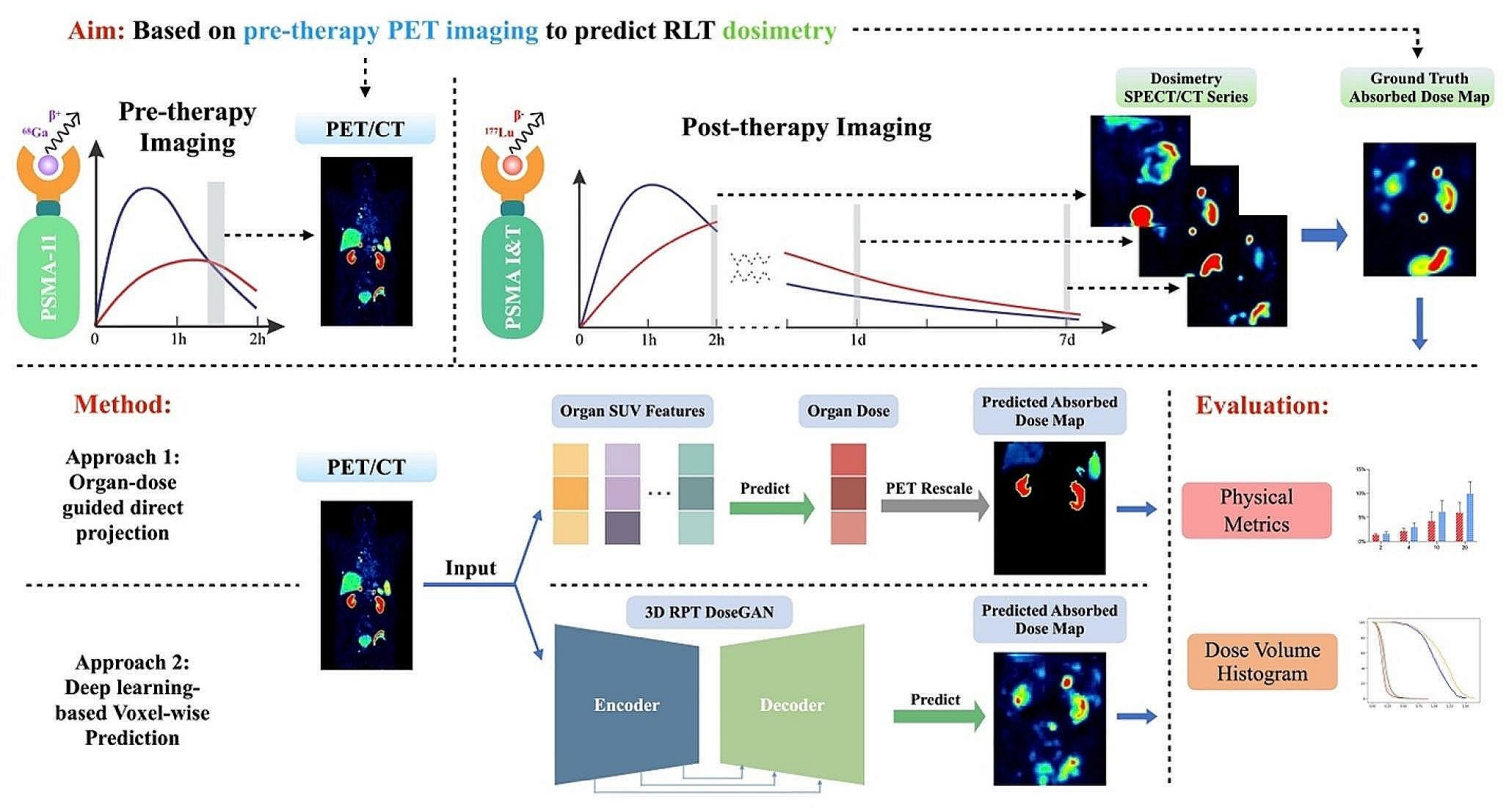
An illustration of our theranostics workflow

We applied the same scheme as our previous developed organ-dose guided pre-therapy prediction for dosimetry with ML-based methods [11]. This approach was developed using SUV features from PET imaging as input, with the corresponding dosimetry of the targeted organ as the ground truth. The PET imaging was rescaled based on the predicted organ dose to generate the dose map (Fig. 2).

#### Approach 2: deep learning-based voxel-wise prediction

Due to limited training samples, we adopted a patch-based approach by extracting 3D image patches of size 32 × 32 × 32 from each image, instead of using the entire 3D image directly, to ensure robustness and reliability of the results. The objective of our model is to predict a dose map, from a pre-therapy PET image. Our proposed 3D RPT DoseGAN comprises two interconnected networks (Fig. 2), namely the generator network and discriminator network, which were initially introduced in [22]. Detailed description of network architecture and training procedures can be found in Supplementary materials (Figure S1).

### Evaluation

#### Evaluation based on physical metrics

To evaluate the prediction uncertainty of our 3D RPT DoseGAN, we measured the voxel-wise deviation between the predicted dose maps and the ground-truth maps. We employed Normalized Root Mean Squared Error (NRMSE) and Structural Similarity Index Measurement [23] as our evaluation metrics. To compare our predictions with 3D RPT DoseGAN to the organ-dose guided method, we manually segmented the OAR, and calculated the NRMSE and SSIM within OAR. Additionally, we measured the deviation of the mean absorbed organ dose using the mean absolute percentage error (MAPE). The ground-truth dose maps were considered as the reference.

#### Evaluation with dose volume Histogram

Dose volume histograms (DVHs) are commonly used in external-beam radiation therapy to summarize and characterize dose distributions [24]. Accurate DVHs can help physicians improve the quality of a treatment plan [25]. Therefore, we also evaluated the accuracy of the DVHs generated from the predicted dose map using the coefficient of determination (R^2^) [26].

## Results

### Voxel-wise correlation between pre-therapy PET and absorbed dose map

**Fig. 3:**
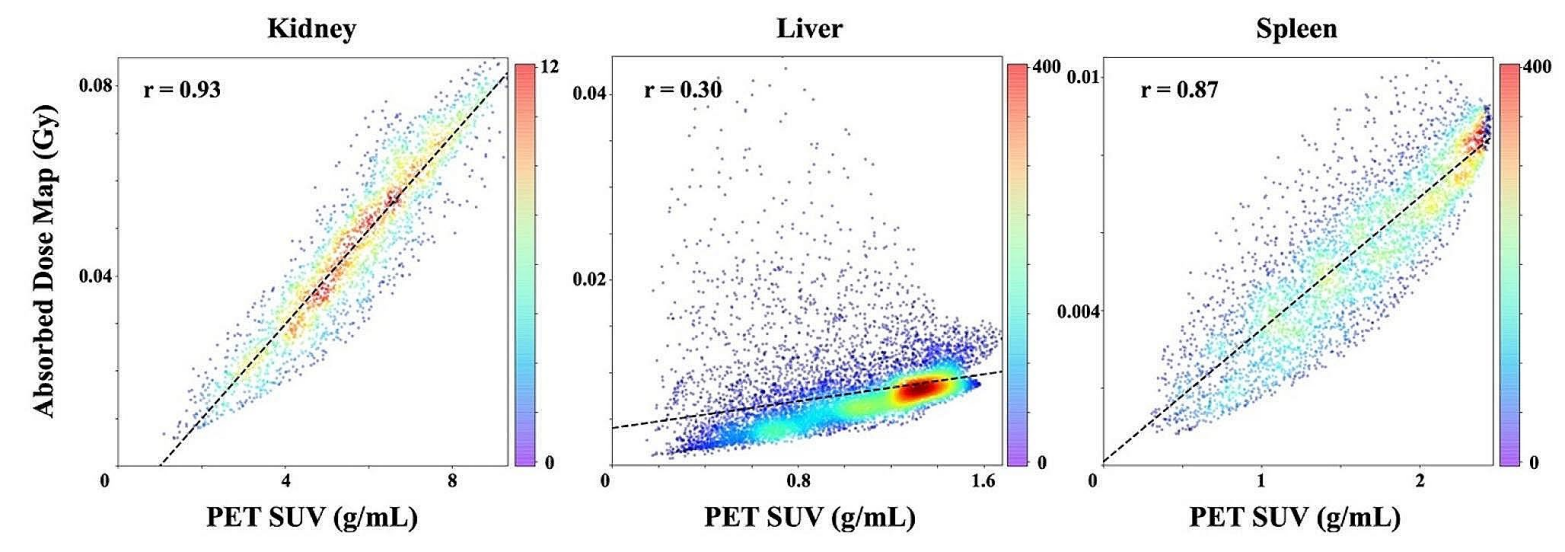
Voxel-wise correlation between PET and absorbed dose map

Moderate correlations were identified between pre-therapy PET and dose map. Figure 3 shows an exemplary scatter heatmap demonstrating the significant correlation between PET and dose map (*P* < 0.025). The mean correlation coefficients (r) of voxel values within each OAR were 0.77 ± 0.14 (mean ± std.) for the kidney, 0.50 ± 0.23 for the liver, and 0.58 ± 0.26 for the spleen. Additionally, Figure S2 demonstrated significant positive correlations of heterogeneity between pre-therapy PET and dose map (*P* < 0.025). The GLRLM had a mean correlation coefficient of 0.71, and the GLCM had a mean correlation coefficient of 0.68.

### Interpretation of the correlation between pre-therapy PET and absorbed dose map

The results of two-tissue compartment model simulation (Figure S3) indicated a significant positive correlation (*r* = 0.99) between PET SUV and dose map solely in scenarios with homogenous pharmacokinetics. However, in cases where the pharmacokinetics differ, two correlation clusters (*r* = 0.96) were observed, and a greater variety of kinetic parameters resulted in a weaker correlation (*r* = 0.40).

### Evaluation

#### Visual comparison of two pre-therapy prediction approaches

**Fig. 4:**
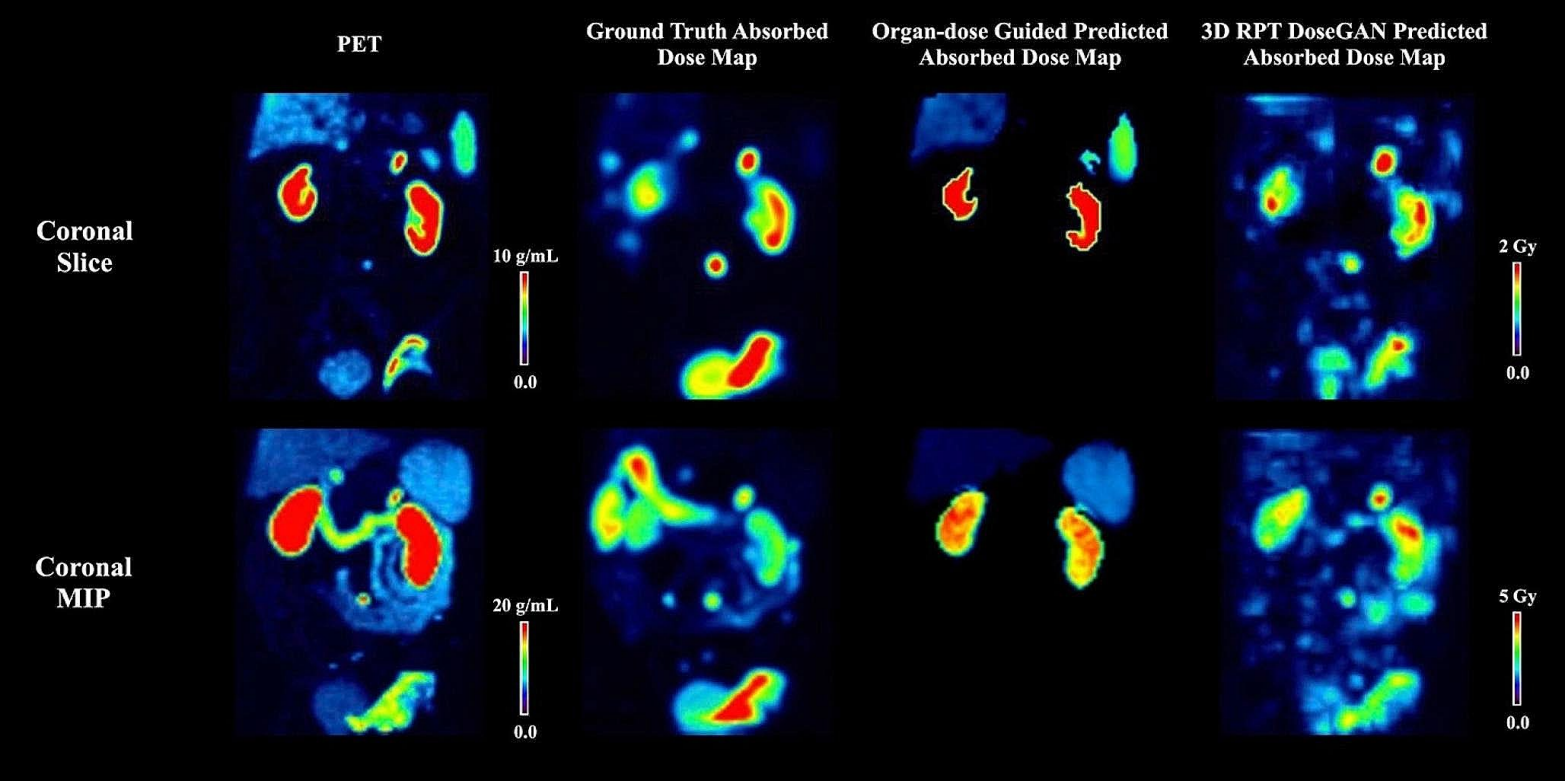
Pre-therapy PET images, absorbed dose map generated by Hermes Voxel-dosimetry tool (Ground Truth), and dose map predicted by organ-dose guided method and our proposed 3D RPT DoseGAN are presented

As confirmed by an exemplary visual reading in Fig. 4, our proposed approach demonstrated good agreement with the ground truth, exhibiting a similar dose distribution profile in organs at risk such as the liver and kidneys, while showing slight overestimation of dose in the spleen. Furthermore, upon reviewing the entire test dataset, our board-certified nuclear medicine physicians confirmed the image quality of our proposed approach for potential clinical use.

**Fig. 5:**
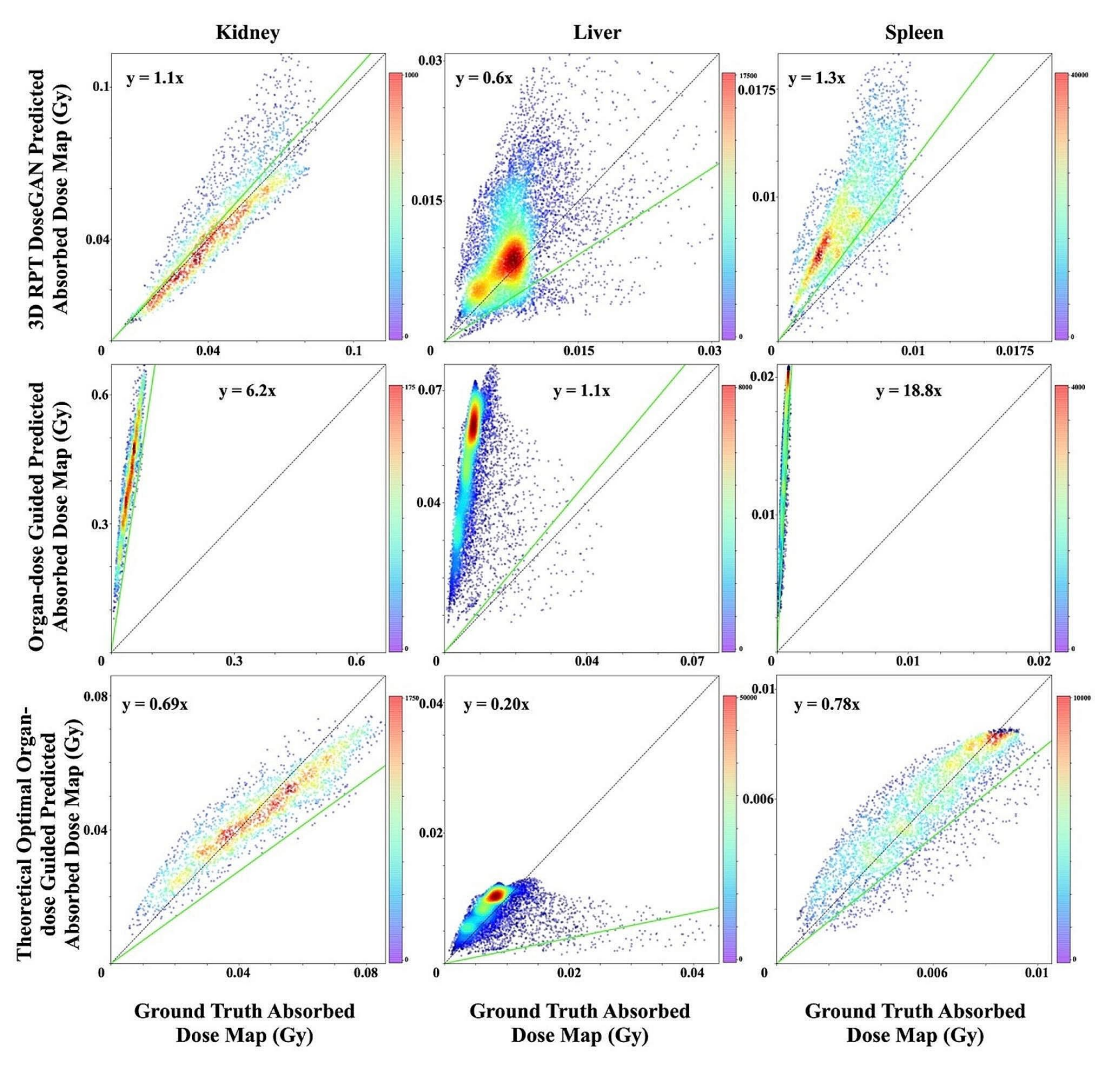
Identity plots of an exemplary subject depicting the correlation between ground-truth and predicted absorbed dose map

The identity plot of an exemplary subject (Fig. 5) demonstrated voxel-wise similarity between the ground-truth dose map and the dose map predicted by 3D RPT DoseGAN. The slopes of the identity lines for kidney, liver, and spleen were 1.1, 0.6, and 1.3, respectively, indicating superior performance compared to the organ-dose guided approach. Even after rescaling based on the mean dose, the theoretical optimal results still exhibited smaller slopes. Statistically, the 3D RPT DoseGAN outperformed the theoretical optimal results of the organ-dose guided approach across all organs in terms of both the slope of the fitting lines and the correlation coefficient (Figure S5), with the mean improvement of slope 24.3% for kidney, 199.2% for liver and 137.1% for spleen.

#### Evaluation based on physical metrics

Our 3D RPT DoseGAN achieved a voxel-wise NRMSE of 1.7 ± 0.4% and SSIM of 0.94 ± 0.03 compared to the ground-truth dose map, the cross-validation results of the DoseGAN are illustrated in Figure S6. At the organ level, as illustrated in Figure S7, the 3D RPT DoseGAN outperformed the organ-dose guided method across all organs at various physical metrics. In particular, for the kidney, our 3D RPT DoseGAN achieved an NRMSE of 0.8 ± 0.1% and an MAPE of 10.8 ± 8.4% compared to the ground-truth dose map, whereas the organ-dose guided approach attained an NRMSE of 1.7 ± 0.3% and an MAPE of 17.6 ± 16.1%.

#### Evaluation with dose volume histogram

**Fig. 6:**
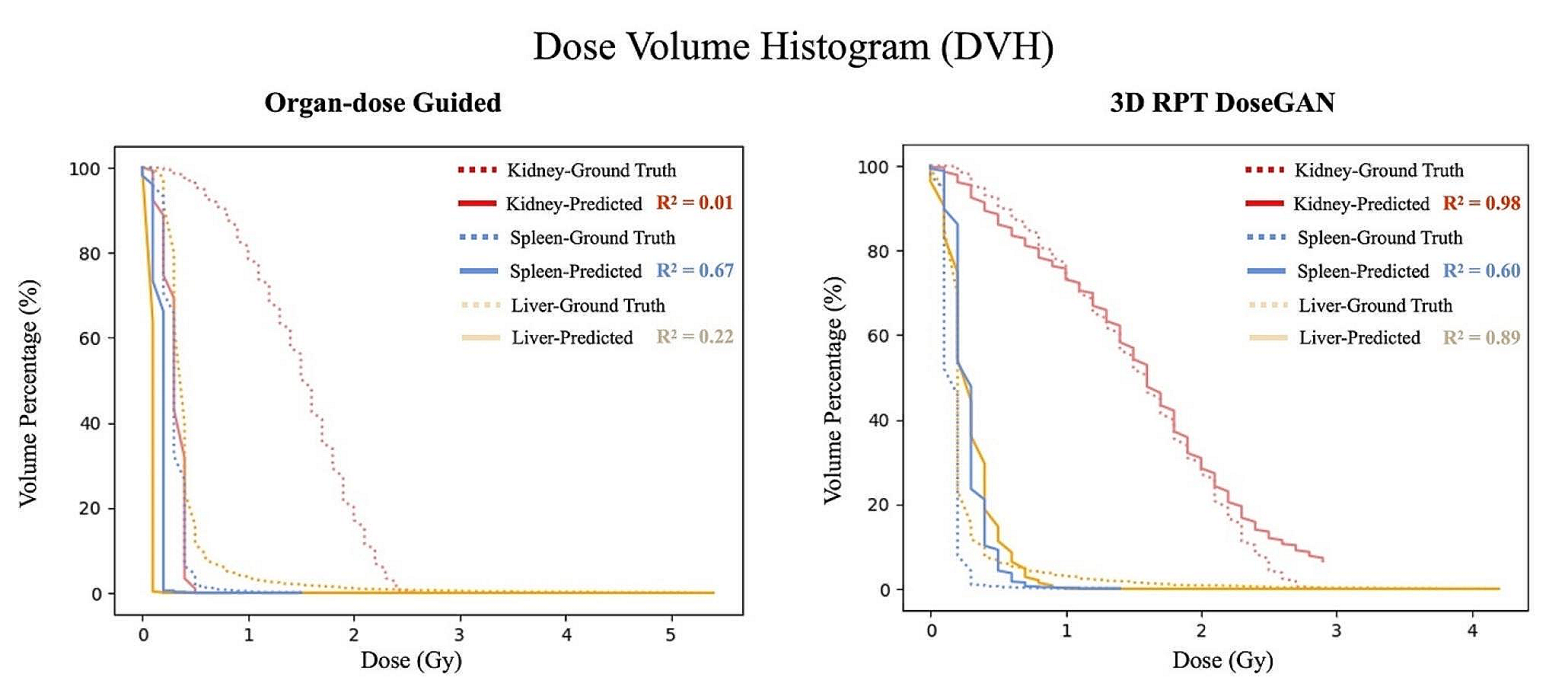
Dose volume histogram (DVH) were plotted to characterize dose distributions of predicted absorbed dose map of organ at risk

Figure 6 shows the ground-truth and predicted DVH curves for each OAR as a plan evaluation example using the organ-dose guided approach and 3D RPT DoseGAN. The R^2^ values of the DVH revealed the better prediction accuracy of 3D RPT DoseGAN. Among all test cases, the kidney showed a strong linear correlation between predicted and ground-truth DVH data points, with mean R^2^ values of 0.92, indicating that the predicted DVH curve nearly overlapped the achieved DVH curve. The mean R^2^ values achieved were 0.74 for the liver and 0.50 for the spleen, respectively. All R^2^ results of 3D RPT DoseGAN significantly outperformed the organ-dose guided approach (*P* < 0.025).

## Discussions

This study aimed to characterize the intra-organ heterogeneity in pre-therapy PET and post-therapy dosimetry, and assess the feasibility of voxel-wise dosimetry prediction. Although prior research has demonstrated a correlation between pre-therapeutic SUV and absorbed dose at the organ level [17], this correlation has not been explored at the voxel level. Given the spatially heterogeneous distribution of radiopharmaceuticals, resulting in uneven energy deposition, accurate characterization is essential in treatment planning. Our investigation unveiled a moderate correlation between the distribution of pre-therapy PET and dose map. Simulation results (Fig. 1&S3) indicated a significant positive correlation (*r* = 0.99) between PET SUV and absorbed dose, assuming proportional kinetic parameters for [^177^Lu]Lu-PSMA I&T and [^68^Ga]Ga-PSMA-11. The suboptimal correlation likely arises from differing kinetics in sub-tissues within each organ, as demonstrated by two correlation clusters (*r* = 0.40) representing varying kinetic parameters of these sub-tissues.

The organ-dose guided direct projection requires solely the contours of targeted organs from pre-therapy PET imaging and the corresponding SUV features extracted from them. These processes can now be accurately performed using automated tools [27]. rendering this approach practical in real-world applications. Nevertheless, this method falls short in capturing the dose distribution owing to intra-organ heterogeneity. In contrast, our proposed 3D RPT DoseGAN demonstrates superior prediction accuracy and effectively unveils this heterogeneity. This improvement can be attributed to the implicit extraction of tissue-specific kinetics by deep neural networks from pre-therapeutic PET, enabling the estimation of kinetics for therapeutic dosimetry. By bridging the gap in intra-organ theranostic heterogeneity, our 3D RPT DoseGAN plays a crucial role in determining the radiobiological effect of the treatment. However, it is essential to validate this hypothesis through further pre-clinical studies on a microscopic scale that may not be discernible with clinical imaging techniques. For instance, as shown in Fig. 3, poorer correlations were observed in the liver, possibly due to a larger variety of pharmacokinetics in the sub-tissues. Additionally, although preliminary retrospective analyses indicate that dosimetry imaging could predict prostate-specific antigen (PSA) response [28], the prognostic imaging biomarker related to overall survival (OS) has not been fully evaluated [29]. Therefore, the voxel-wise predicted dose map can better assist the development of such biomarkers.

Our study developed a model with only 48 paired theranostic companions, a sample size that may be suboptimal for robust deep learning development. Despite applying augmentation techniques such as patching, the total number of cycles, which is 48, is still insufficient for the development of image synthesis tasks for DL. Additionally, patch-based inputs are highly correlated, which limits the information available for training. Although we attempted to reduce data correlation through random shuffling, a larger dataset would greatly benefit the model in terms of robustness and accuracy. Moreover, the quality of the current dataset has diminished the advantages of voxel-wise dosimetry, as the spatial resolution and field of view of the dosimetry SPECT/CT are limited. We are currently collecting data from our own center using PET with improved sensitivity and resolution (Siemens Vision Quadra) and two different tracers (^68^Ga and ^18^F labeled PSMA). Additionally, dosimetry SPECT/CT data with a larger field of view, covering beyond the abdominal region, are being collected. This will enable the prediction of doses for more organs at risk, such as the salivary glands. Furthermore, with the availability of pre-therapy dynamic PET data, models can be developed to predict series of SPECT. Alternatively, with post-therapy PET data, reinforcement learning techniques can be applied to optimize the developed models.

Another bottleneck in our study is the quality of the ground-truth data used for development. Conventionally, voxel-wise dosimetry is conducted using techniques such as voxel S-value (VSV) [13], dose point kernel (DPK) [30], and Monte Carlo (MC) simulation [31]. VSV is a voxel-level implementation of the Medical Internal Radiation Dose (MIRD) schema, which defines S-values. DPK, applied by Siemens Dosimetry Research Tool, measures the absorbed energy per unit mass in a homogeneous water phantom to define radial absorbed dose [32]. However, both VSV and DPK are limited by their reliance on homogeneous phantoms. MC simulation is a more accurate personalized dosimetry technique that can be applied to heterogeneous activities and media. The “semi” Monte Carlo (sMC) simulation, applied by Hermes Voxel-dosimetry tool, simulates and tracks particles at the voxel-level to estimate absorbed dose by calculating accumulated activity [33]. However, MC simulation is computationally demanding, time-consuming, and often cumbersome to set up, despite efforts by Hermes to accelerate its tool using simplified methods [34]. Alternative methods should be explored in further studies due to the limitations of DPK and sMC simulation in terms of heterogeneity issues or computational time and resource requirements. Furthermore, both MC simulation and DPK rely on SPECT and CT images from multiple time points as input, assuming that patients and organs do not move during PET or SPECT imaging. However, patient motion during imaging is inevitable and can result in artifacts and reduced image quality [35], which consequently affect the accuracy of the dose map used as our ground truth. Additionally, the procedure of co-registration between pre-therapy PET images and dose map images was limited to rigid registration due to the absence of mature deformable registration tools, which may introduce inaccuracies due to changes in patient weight or soft tissue displacements during breathing [36]. Future studies should consider using advanced registration tools or other possible solutions to improve the accuracy of the ground truth for model training.

## Conclusion

Our preliminary results demonstrated the intra-organ heterogeneity of pharmacokinetics, leading to the difference of the distribution between pre-therapy PET imaging and dose map, which may challenge the pre-therapy prediction of dosimetry. DL has the potential to bridge this gap for pre-therapy prediction of voxel-wise heterogeneous dose map. The experimental findings in the present work provide the evidence that our proposed DL approach, i.e. 3D RPT DoseGAN is capable to capture the dosimetry heterogeneity using pre-therapeutic PET imaging. This advancement has the potential to accelerate the implementation of dosimetry-guided treatment planning for RPT, leading to improved therapeutic efficacy, reduced adverse events, and ultimately better patient outcomes.

### Electronic supplementary material

Below is the link to the electronic supplementary material.

Supplementary Material 1

## Data Availability

No.
