# Supplementary material for: Pre-therapy PET-based voxel-wise dosimetry prediction by characterizing intra-organ heterogeneity in PSMA-directed radiopharmaceutical theranostics
Source: Eur J Nucl Med Mol Imaging. 2024 May 9;51(11):3450–60. doi: 10.1007/s00259-024-06737-3 (PMC11368979; doi:10.1007/s00259-024-06737-3)
Supplement: Supplementary file 1 — Supplementary Material 1 [file 259_2024_6737_MOESM1_ESM.doc]

**Characterize Intra-organ Heterogeneity in PSMA-directed Radiopharmaceutical Theranostics for Voxel-wise Pre-therapy Dosimetry Prediction**

Song Xue^1^, Andrei Gafita^2^, Yu Zhao^3^, Fangxiao Cheng^1^, Isabel Rauscher^2^, Calogero D'Alessandria^2^, Ali Afshar-Oromieh^1^, Axel Rominger^1^, Matthias Eiber^2^, Kuangyu Shi^#1,3^

1. Dept. Nuclear Medicine, Bern University Hospital, University of Bern, Switzerland
2. Dept. Nuclear Medicine, Technical University of Munich, Germany
3. Chair for Computer Aided Medical Procedures, School of Computation, Information and Technology, Technical University of Munich, Germany

^#^Corresponding:

Prof. Kuangyu Shi,

Department of Nuclear Medicine

Inselspital, Bern University Hospital,

University of Bern

Bern, Switzerland

Email: [kuangyu.shi@unibe.ch](mailto:lb10363@rjh.com.cn)

**Materials and Methods**

**Pre-therapy Prediction of Absorbed Dose Map**

**Approach 2: Deep learning-based Voxel-wise Prediction**

The generator network aims to predict the dosimetry distribution map given a pre-therapy PET image as input. Considering that both pre-therapy PET images and dosimetry map images are representations of the same underlying structure, they share a significant amount of low-level information. Therefore, we designed the generator network using a U-net like architecture, which incorporates skip connections to transfer low-level feature representations directly from encoding layers to decoding layers (1). The discriminator network contributes an additional adversarial loss to the model, which encourages the predicted dosimetry image to closely resemble the real dosimetry image.

The training procedure follows the standard approach as described in (2), and we utilized the Adam solver with a batch size of 4 and a learning rate of 0.0002. To efficiently access the large number of images during training, we organized the dataset into a single data object in HDF5 (Hierarchical Data Format 5). During testing, the input PET patches from the test dataset were fed into the trained generator network, and the corresponding dosimetry images were predicted patch-wise and subsequently assembled together. All experiments were implemented using TensorFlow and Keras, and were trained on NVIDIA GeForce GTX 1080 Ti graphics cards for optimal performance.


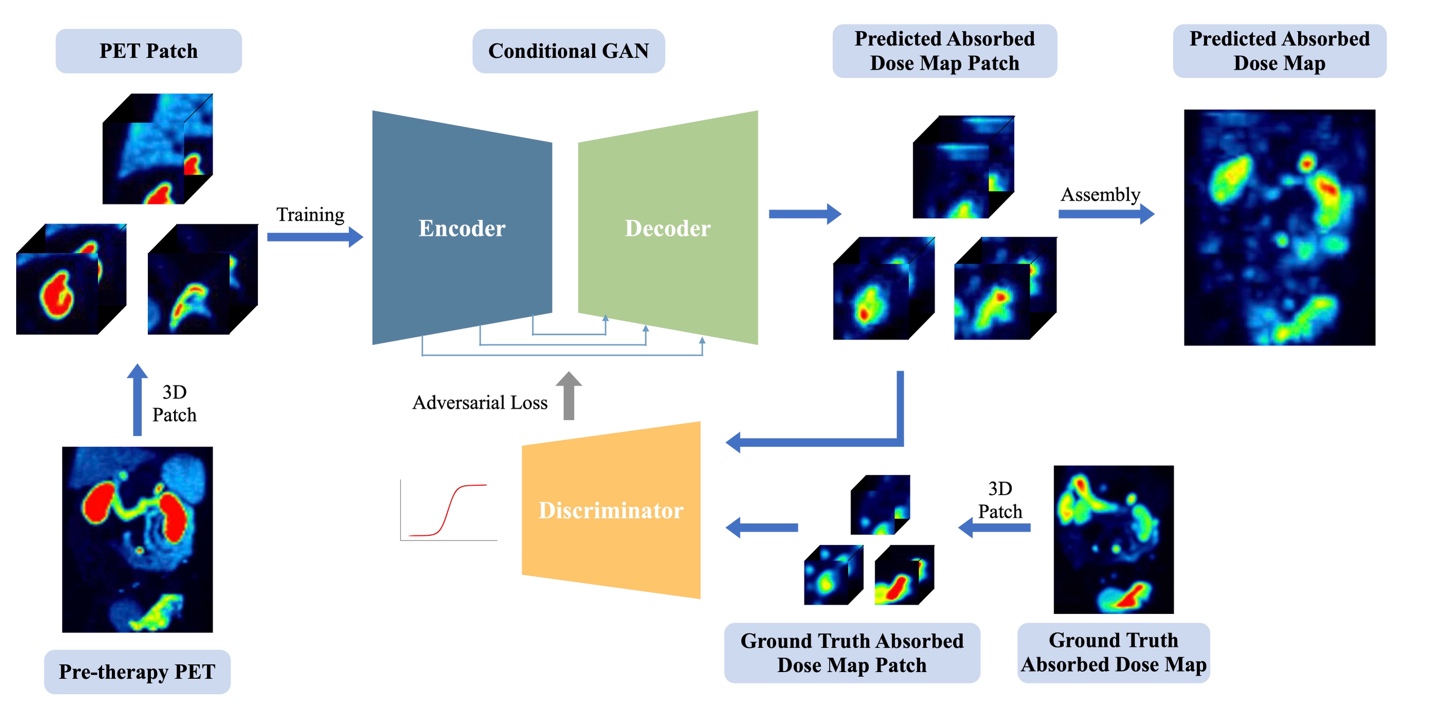


**FIGURE S1.** Detailed illustration of our proposed 3D RPT DoseGAN.

**Evaluation**

**Evaluation based on physical metrics**

To evaluate the prediction uncertainty of our 3D RPT DoseGAN, we measured the voxel-wise deviation between the predicted dosimetry images and the ground-truth images. We employed Normalized Root Mean Squared Error (NRMSE) and Structural Similarity Index Measurement (SSIM) as our evaluation metrics. The NRMSE is defined as:


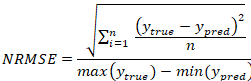


where
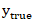
 is the ground truth dosimetry image and
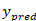
 is the predicted one. It is important to note that while voxel-wise quantities such as NRMSE are straightforward to calculate and compare, they may not necessarily correspond well with the errors perceived by humans, and images with similar NRMSE values may still appear substantially different to the human eye (3). Therefore, additional measures such as SSIM, which more accurately reflect perceptual differences, are desirable. The SSIM is defined as:


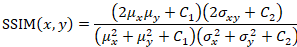


where
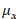
,
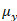
 are the averages of images
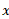
 and
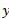
, and
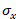
,
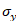
 are their standard deviations, respectively.
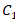
 and
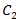
 are two positive constants to avoid a null denominator. Theoretically, lower NRMSE and higher SSIM values indicate lower prediction uncertainty.

**Results**

**Voxel-wise Correlation between Pre-therapy PET and Absorbed Dose Map**

**
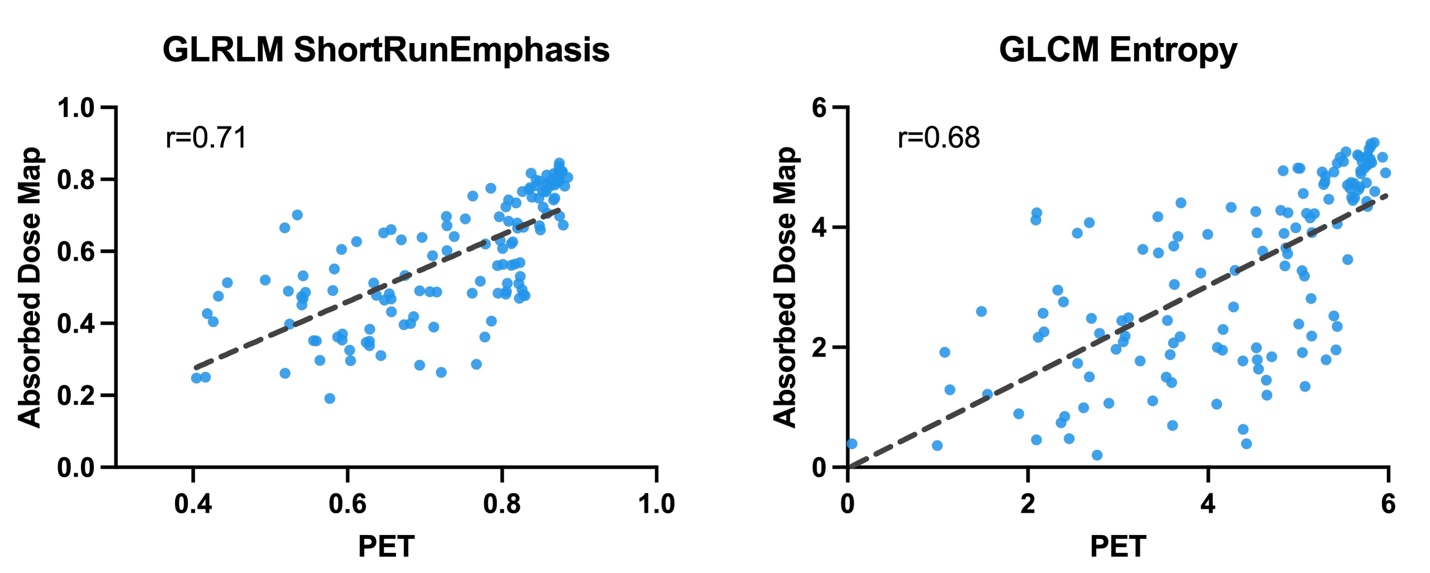
**

**FIGURE S2.** Correlation of heterogeneity between PET and absorbed dose map.

**Theoretical interpretation of the Correlation between Pre-therapy PET and Absorbed Dose Map**


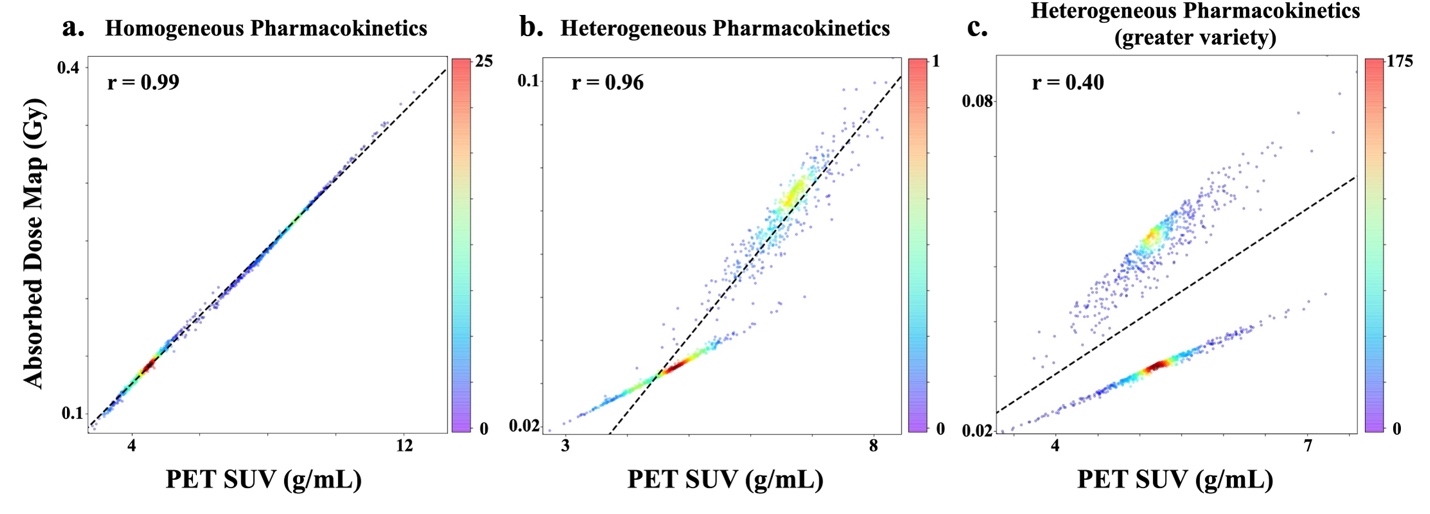


**FIGURE S3.** Voxel-wise correlation between simulated pre-therapy PET and absorbed dose map (ADM).

**Evaluation**

**Visual Comparison of Two Pre-therapy Prediction Approaches**

As depicted in the heatmap of voxel-wise difference of dose distribution in each organ (Figure S4), our method not only demonstrated superior prediction accuracy compared to the organ-dose guided prediction, but also effectively revealed the heterogeneity of the dose distribution.


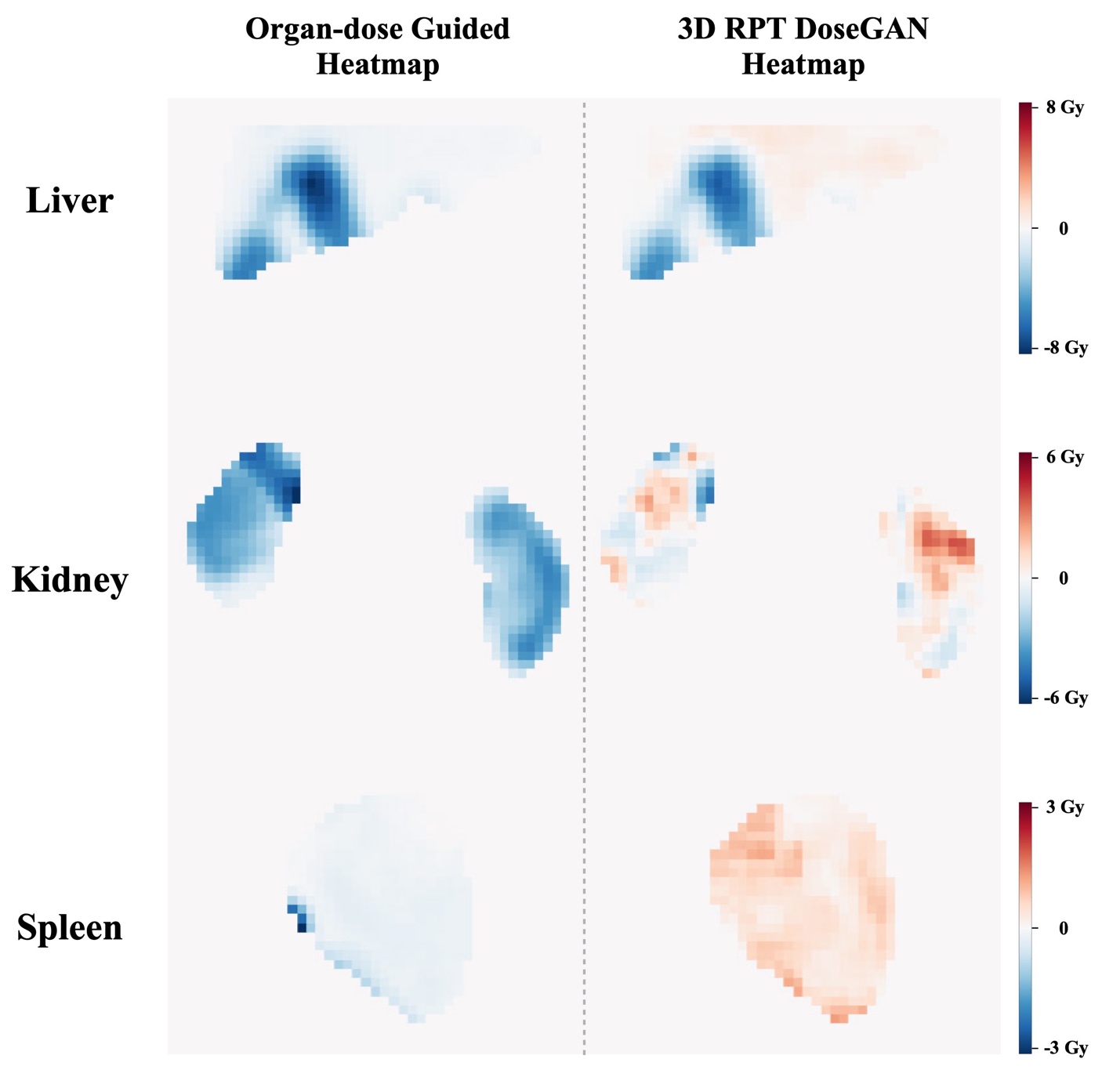


**Figure S4.** Heatmap of each organ at risk in terms of the voxel-wise difference regarding to ground-truth dosimetry images.


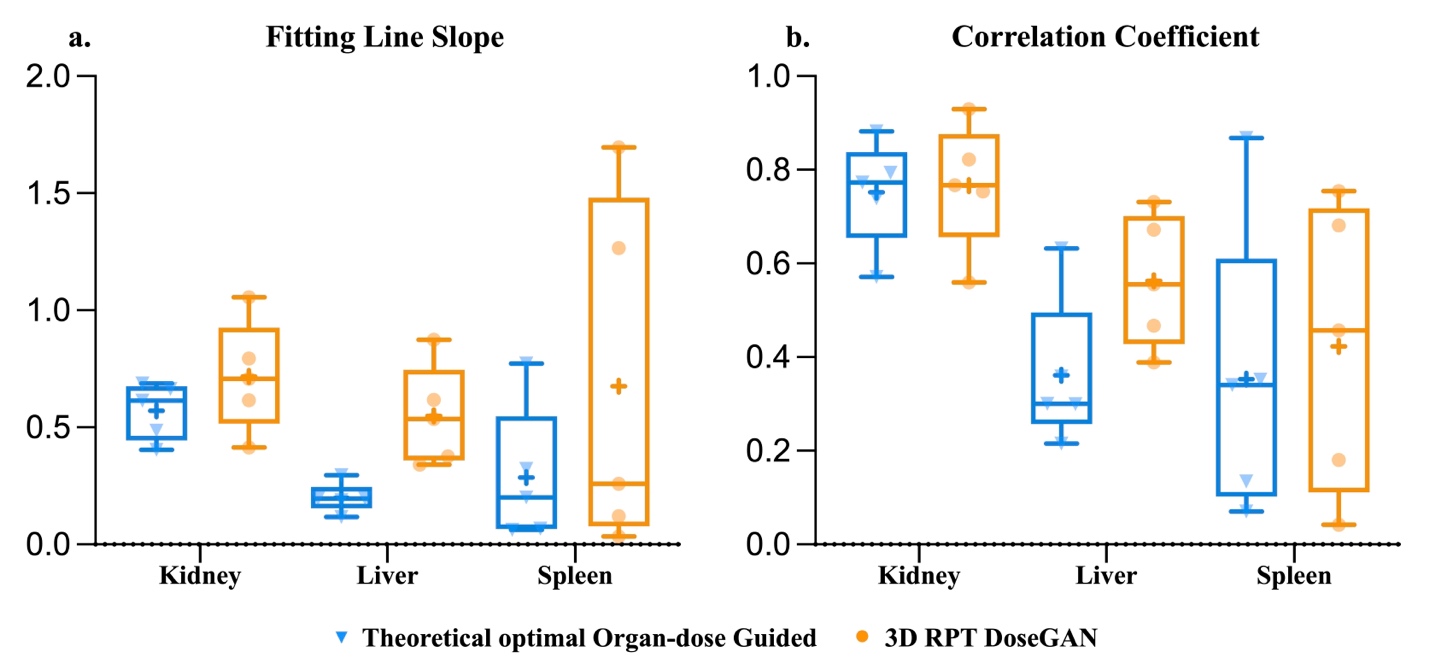


**Figure S5.** Statistic results of identity plots, with fitting line slope and correlation coefficient (r).

**Evaluation based on physical metrics**


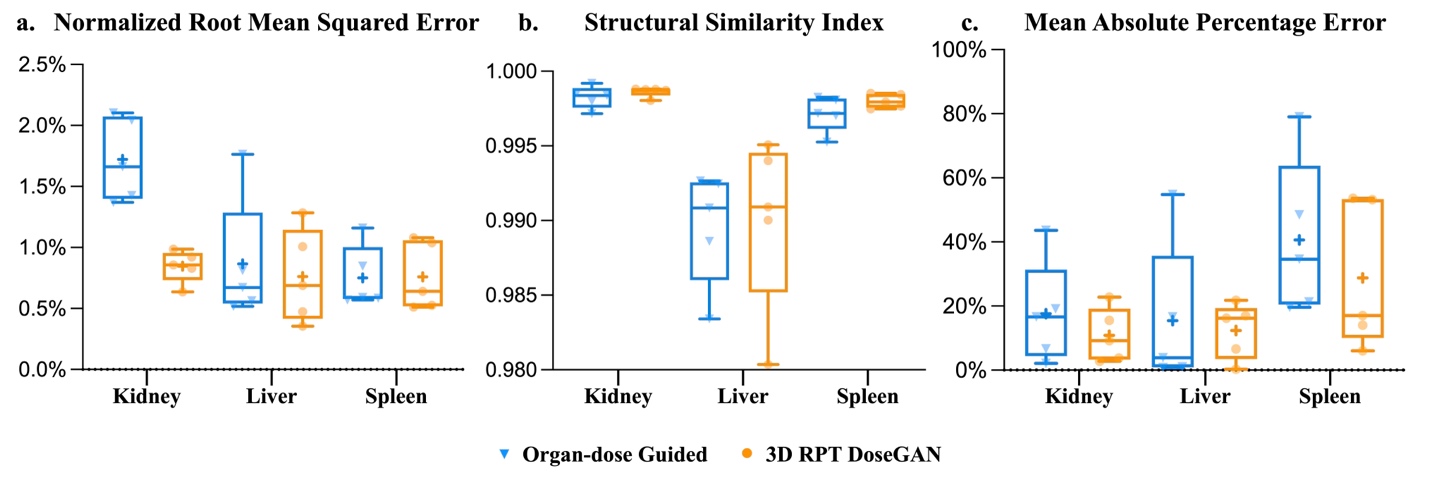


**Figure S6.** Quantitative accuracy evaluated with NRMSE, SSIM and MAPE within organ at risks.

**Reference**

1. Ronneberger O, Fischer P, Brox T. U-net: Convolutional networks for biomedical image segmentation. International Conference on Medical image computing and computer-assisted intervention: Springer, 2015; p. 234-241.

2. Goodfellow I, Pouget-Abadie J, Mirza M, Xu B, Warde-Farley D, Ozair S, Courville A, Bengio Y. Generative adversarial nets. Advances in neural information processing systems2014; p. 2672-2680.

3. Wang Z, Bovik AC, Sheikh HR, Simoncelli EP. Image quality assessment: from error visibility to structural similarity. IEEE transactions on image processing 2004;13(4):600-612.
